# The Evolutionary-Ecological Basis of Xenophilia

**DOI:** 10.1007/s12110-026-09521-4

**Published:** 2026-05-04

**Authors:** Cyril C. Grueter

**Affiliations:** 1 Institute of Human Sciences, School of Anthropology and Museum Ethnography, University of Oxford, Oxford, UK; 2 International Centre for Biodiversity and Primate Conservation, Dali University, Dali, Yunnan China; 3 Department of Anatomy, Physiology and Human Biology, School of Human Sciences, The University of Western Australia, Perth, WA 6001 Australia; 4 Centre for Evolutionary Biology, School of Biological Sciences, The University of Western Australia, Perth, WA 6001 Australia; 5 Centre of Excellence in Biodiversity and Natural Resource Management, University of Rwanda, Kigali, Rwanda

**Keywords:** Inter-group tolerance, Peace, Trade, Exogamy, Collective defence, Primates

## Abstract

Humans are uniquely adept at cultivating positive, xenophilic relationships beyond their immediate group, forming bonds through various mechanisms such as trade, exogamy, and shared defense. Despite the significance of intergroup cooperation, synthetic approaches to understanding the evolutionary, ecological, and institutional drivers of xenophilia remain underdeveloped compared to the wealth of research on intergroup conflict. Here, I synthesize ethnographic, archaeological, psychological and comparative evidence to argue that xenophilia is supported by recurrent human cognitive and social capacities whose expression and stability depend on ecological, economic and institutional contexts. The aims of this paper are threefold: first, to clarify the selective pressures and incentives that favour peaceful intergroup relations, with particular attention to factors contributing to amicable intergroup ties and expanding the circle of tolerance and cooperation, including economic interdependence through trade and reciprocity, information exchange, exogamy, and the necessity of collective defense against external threats; second, to examine how these different drivers of xenophilia interact and under what conditions they generate, stabilize or fail to sustain intergroup tolerance; and third, to place human xenophilia in comparative perspective by highlighting both parallels and distinctions with non-human primates in their strategies for fostering intergroup peace. By distinguishing between mechanisms that generate cooperation and those that stabilize it in the face of freeriding and defection, this synthesis highlights how humans uniquely scale and institutionalize intergroup peace. This perspective is particularly timely, as uncovering the evolutionary roots of tolerance and cooperation across group boundaries remains crucial for addressing pressing global challenges such as climate change, poverty, famine, conflict, and disease.

## Introduction

While parochial motivations are widespread in humans and probably have a long evolutionary history (Brewer, 2010; Choi & Bowles, 2007; Romano et al., 2021), there is substantial variability in how strongly such motivations are expressed (Pisor & Ross, 2023). Strong in-group favouritism and group identity effects can be loosened, giving rise to cooperative or prosocial behaviour toward out-group members (Fry, 2006; Glowacki, 2024; Grueter & White, 2014; Hames, 2019; Meijer, 2024; Pisor & Surbeck, 2019). These cooperative interactions take many forms, including the exchange of goods, services, and information; the formation of marriage alliances; ritual gatherings; and coordinated defense against common threats. Some forms of xenophilia appear to be evolutionarily ancient, as evidenced by long-distance transport of raw materials indicating supralocal exchange networks by at least 300,000 years B.P. (Brooks et al., 2018).

In small-scale societies, i.e. those characterized by relatively low population density, subsistence-oriented economies, and weak market integration, cooperative and affiliative activities occur at both smaller and larger social scales. Cooperative hunting (Alvard & Nolin, 2002; Hill, 2002; Steward, 1938), labor exchange in cultivation (Hames, 1987; Moore, 1975), food sharing (Dyble et al., 2016), and cooperative child rearing (Hrdy, 2009) typically bond members of lower-level social units such as families and households. At the same time, many foraging and horticultural societies engage in large-scale cooperation to produce collective goods, including mass hunting events, large labor mobilizations, and shared infrastructure projects whose success depends on recruiting a critical mass of participants (Boyd & Richerson, 2022; Ember et al., 2018; Singh & Glowacki, 2022). Archaeological and ethnographic evidence suggests that such large-scale cooperative undertakings were also present among nomadic societies of the Holocene and Pleistocene (Ortmann & Kidder, 2013; Rodriguez-Hidalgo et al., 2017).

Humans feature some characteristics that serve as preconditions for the evolution of such a sophisticated form of intergroup peace. The level of encephalization needed for the effective management of intergroup relationships is clearly in place in humans. Other important cognitive mechanisms are the ability to mentalize the states of others and to engage in collaborative activities with shared goals and intentions (Chapais, 2013; Glowacki, 2024; Tomasello et al., 2005). Recent work further suggests that selection in humans has favored cognitive capacities for providing “computational services” to others (such as social inference, coordination, mediation, and information integration) which allow these social-cognitive abilities to be deployed beyond dyadic interactions and to scale cooperation within and between groups (Hagen et al., 2025). Another facilitator of interactions across group boundaries is an effective language-based communication system (Benítez-Burraco & Kempe, 2018; Fuentes, 2009; Wilson et al., 2017). For example, the functioning of large-scale exchange networks relies on a communication system that encompasses syntactic structure, the socially contextual use of symbols, and the capacity to convey information about non-immediate entities or events—core features that characterize human language (Marwick, 2003). An additional evolutionary factor that may have promoted more harmonious intergroup relations is self-domestication, a process in which selection against reactive aggression fostered greater social tolerance and was accompanied by morphological changes such as reduced craniofacial robusticity and smaller teeth (Cieri et al., 2014; Wrangham, 2019). Psychologists have also suggested that a secure and valued sense of group membership, in particular that characterized by identity fusion, may provide individuals with the psychological resources necessary to initiate and sustain trustworthy, cooperative relationships across group boundaries (Klein & Bastian, 2023; Klein et al., 2024). However, the expression of xenophilic inclinations can also vary among individuals due to individual dispositions such as personality, e.g. those that score higher on openness to experience are more inclined to invest their personal resources in exploring opportunities for cross-group interactions (Danckert et al., 2017; Stürmer et al., 2013). Together, these capacities help explain why xenophilic interactions are not only possible in humans but can generate substantial fitness-relevant benefits under a wide range of social conditions.

We briefly review these benefits not because they are theoretically controversial but to clarify the selective pressures that would favour the evolution and maintenance of xenophilic capacities. Humans can derive a large number of benefits from peaceful intergroup interactions (Glowacki, 2024). Several variables have been identified as contributing to amicable intergroup ties and facilitating the expanded circle of tolerance and cooperation in humans, including information exchange, exogamy, economic interdependence (trade and reciprocal exchange of goods), and the need for common defence against external adversaries (Alexander, 1990; Chapais, 2011; Fry, 2012; Grueter & White, 2014; Hill et al., 2011; Pisor & Surbeck, 2019; Rodseth et al., 1991). When the interests of group members align, e.g., in scenarios where collective defense against common rivals is essential, the benefits of xenophilia can extend to all group members. However, there are also situations where intergroup connections are more limited, involving only a subset of members from each group, such as those linked through marital ties.

Yet compared to the extensive work on intergroup conflict, the benefits of xenophilia have received relatively little systematic attention. In this paper, we bring together ethnographic, archaeological, psychological, and primatological evidence to suggest that xenophilia is a recurrent and adaptive feature of human social life. Our contribution is threefold: to highlight the diverse benefits of peaceful intergroup interactions; to examine how different drivers of xenophilia interact (e.g. trade, exogamy, information exchange, collective defense), while also distinguishing between human-unique processes that primarily stabilize existing cooperative relationships and those that constitute qualitatively distinct pathways to intergroup tolerance; and to situate human xenophilia in comparative perspective by assessing its analogues and limits in non-human primates. Rather than treating xenophilia as a rare or anomalous outcome, this paper focuses on the recurrent human capacities and mechanisms that make peaceful intergroup relations possible, while recognizing that their expression and durability are contingent on ecological, economic and institutional contexts. By foregrounding the evolutionary and ecological payoffs of xenophilia, we aim to complement existing research on intergroup relations and add to ongoing discussions of how cooperation across group boundaries has shaped human social evolution.

## Economic Interdependence

Pisor and Surbeck (2019) nominated the need to buffer local resource losses and gain access to non-local resources in the human foraging ecology as a prime candidate for the formation of extra-group tolerance and the integration of groups into larger-scale symbiotic networks. In environments where resources are spatially heterogeneous and/or temporally asymmetric between groups, access to resources outside one’s home turf can be achieved by weaving webs of relationships with other communities. These exchange networks function as cushioning mechanisms when their local resources are hard-hit by environmental disturbance and as tickets to desired, yet locally unavailable, comestibles^1^ (Cashdan, 1983; Kelly, 2013; Pisor & Gurven, 2016; Roberts, 2005; Silberbauer, 1972; Spielmann, 1986; Sterelny, 2014; Wiessner, 2002). In the face of climate change, such social investments aimed at alleviating resource shortages may become even more important, a topic that needs more empirical testing (Pisor & Jones, 2021).

Proximately, this nexus of economic interactions can be solidified by symbolic gift exchange. *Hxaro*, the system of delayed reciprocity among the !Kung San people of the Kalahari Desert, is a well-known example (Wiessner, 1982). Another iconic ethnographic illustration of reciprocal gift exchange is observed in the Melanesian *Kula* ring. Within this cultural practice, prized items like shell-disc necklaces are ceremoniously swapped among various island communities, serving to establish and fortify harmonious connections among individuals (Malinowski, 1920).

There is ample ethnographic and archaeological evidence of reciprocal access to dispersed resources as well as exchange of resources across locales. For example, the communities inhabiting the vast expanse of the Great Western Desert in Australia lived in interconnected bands, forming local groups with shared access rights to vital resources like water and food (Tonkinson, 2004; see also Fry, 2006). Similarly, during years of reduced rainfall, multiple! Kung camps in the Kalahari merged into larger groups to share water resources at communal water holes (Lee, 1972). In a hunter-fisher-gatherer society in Tierra del Fuego, various communities exploited stranded whales and reciprocated by publicly announcing their find and granting others access to their territory (Santos et al., 2015). In African pastoral systems, reciprocal exchange networks involving livestock have been identified as a major social risk-management strategy that contributes to social integration (Moritz et al., 2011; Spencer, 1973). Among Mongolian pastoralists, temporal unpredictability in productivity of pastures favours fuzzy group boundaries (Fernandez-Gimenez, 2002). In present-day Madagascar, the Masikoro, who are farmer-herders, rely heavily on Vezo merchants, who are fishers, for a significant portion of their protein intake, which primarily comes from marine sources. Similarly, the Vezo community depends on the Masikoro for carbohydrate-rich staples like manioc and maize (Tucker et al., 2021). The Hupa of California engaged in exchanges of commodities with the Yurok inhabiting the coast. In exchange for dried seaweed, the Hupa provided the coastal residents with acorns and various other foods from the inland regions (Wallace, 1978).

Archaeological evidence from prehistoric times paints a similar picture. In late prehistoric northern Chile, communities residing across various ecological zones in the region actively participated in extensive trade networks. This facilitated the mitigation of risks associated with localized shortages and guaranteed access to essential resources from distant areas (Zori & Brant, 2012). Lastly, Stewart et al. (2020) present findings suggesting long-distance, macro-scale exchange ostrich eggshell beads across ecologically complementary regions among African foragers from highland Lesotho that stretch back into the late Middle Stone Age. For groups inhabiting the Karoo scrub and highveld grasslands, acquiring entry to the diverse and resource-abundant upland areas would have encouraged the initiation and upkeep of trading connections with foragers dwelling in the highlands.

While heterogeneity in the distribution and availability of resources can be a potent incentive for peace, temporal unpredictability in resource access does not always promote an open border policy (Pisor et al., 2023). Ember et al. (2018), using ethnographic data from a worldwide sample of 98 societies, present evidence in favour of the theory that customary beyond-household sharing is an adaptive social solution to the risk of resource stress (such as food-destroying natural hazards) in unpredictable environments. However, resource stress does *not* lead to large-scale customary sharing *beyond* the community and beyond relatives. To the contrary, high levels of resource unpredictability or stress including natural disasters can strengthen territoriality (Chiaravalloti & Dyble, 2019) or prompt forcible measures for resource procurement from other groups (Ember & Ember, 1992; LeBlanc, 1999; Zhang et al., 2007). De Dreu et al. (2022) provide experimental evidence that environmental unpredictability and ensuing carrying capacity stress leads to parochial in-group concerns and more out-group aggression.

The link between trade and peace has been explored through comparative analyses as well. A cross-cultural analysis revealed that inter-community food trade in traditional societies is negatively correlated with the extent of conflict between communities, providing support for the idea that trade can act as an inhibitor of conflict between groups (Grueter & White, 2014; but see Tefft, 1975). Economic and ecological interdependence can also have a pacifying function at higher sociopolitical levels (Fry, 2006, 2012; Kupchan, 2010). Economic interdependence can discourage war because it can increase economic utility for a country (Copeland, 2014). Among modern nations, trade is negatively correlated with warfare (Gelpi & Grieco, 2008; Jackson & Nei, 2015). Yet some studies suggest a more complex relationship: while bilateral trade may reduce the risk of conflict by raising opportunity costs, multilateral trade openness can increase the probability of war by weakening specific interdependencies between trading partners (Martin et al., 2008). Trade and resource exchange can not only lead to the acquisition of vital resources and the harmonization of between-group relations, it can also afford status to those well-connected individuals who secure resources for their home community (Braun & Plog, 1982; Pisor & Surbeck, 2019).

The above shows that demand for non-local resources can, under some conditions, motivate the cultivation of neighbourly relations with outsiders, although such demand has also frequently been pursued through conflict and coercion. Whether resource competition gives rise to intergroup cooperation or violence appears to depend less on the resource itself than on pre-existing social, institutional and ecological conditions that shape the feasibility and payoffs of peaceful exchange. However, this line of thinking differs somewhat from studies showing that greater market integration is a powerful correlate of prosociality toward strangers (Baldassarri, 2020; Henrich et al., 2001). One way to reconcile these findings is to distinguish between “push” and “pull” drivers of intergroup cooperation. In contexts of low market integration, cooperation with outgroups is often pushed by necessity, such as the need to buffer risk or access non-local resources. By contrast, in highly market-integrated settings, cooperation is increasingly pulled by opportunity: repeated positive-sum interactions, institutionalized norms governing behaviour toward strangers and reduced risks of exploitation promote more impartial prosociality (Godoy et al., 2005).

If greater market integration means satiation of resource demands, then the need to develop friendly relationships with outgroups may be less pressing. However, under food security, instead of reducing the need to cooperate with out-group members, people are more impartial in their cooperative investments and generally willing to cooperate with out-group members. This may be because in market-integrated societies where strangers regularly engage in positive-sum transactions, there are usually norms that prescribe behaviour toward strangers (Bowles, 1998). Moreover, in societies where basic needs are more easily met – such as market-integrated societies with high levels of material security and institutional safety nets like welfare systems – xenophobia tends to diminish, giving way to more inclusive attitudes toward outgroups (Hruschka et al., 2014).

Trade may not always lead to permanent pacification but may only offer temporary relief from intergroup hostilities. (Mathew, 2022) report for the Turkana pastoralists that periods of negotiated peace between communities, during which grazing and water resources are shared, are usually short-lived. There are abundant historical, ethnographic, and archaeological findings indicating that communities involved in trade often found themselves entangled in occasional bouts of hostile conflict (Junker, 1999; Keeley, 1996; Van der Dennen, 2014). While trade can be a powerful tool for mitigating enmity, trade-offs are not always seen, and cooperative interactions can occur alongside intergroup hostilities in some societies. (Ember et al., 2002) note that conflicts tend to intensify into warfare more often among trading partners compared to nations with limited trade ties. Groups can also maintain peace with one or more other groups while concurrently engaging in conflict with different groups (Glowacki, 2024). Lastly, the adaptiveness of trade is contingent on transaction and opportunity costs. When group-external trade interferes detrimentally with group-internal economic production, trade may become unprofitable. Also when transaction costs are high as a result of e.g. low population density and poor transportation infrastructure, trade can be stymied (Davis, 1966).

## Information Exchange

Extra-community relationships provide a conduit for obtaining socially-transmitted information about the local environment such as resource availability (Fitzhugh et al., 2011) or alternative and more efficient methods of resource acquisition (Henrich, 2016). Information exchange can also spread across different subsistence modes, e.g., when hunter-gatherers adopt technology from agriculturalists (Spielmann & Eder, 1994).

Such information flow is facilitated by the fact that many foraging societies are embedded in large-scale, fluid and unbounded social networks. Individuals routinely move among camps, aggregate seasonally and maintain ties across broad regions, allowing information about resources, technologies, and social conditions to circulate well beyond local groups (Bird et al., 2019). Ultimately, these interactions contribute to broader social cohesion by reducing uncertainty, enabling coordination, and fostering tolerance among groups that are linked through shared informational landscapes.

## Exogamy

It has long been recognized by anthropologists that exogamous behavior, whereby individuals (mostly women) marry outside their natal community, can keep violence at bay and foster cordial ties between distinct groups (Fox, 1983; Lévi-Strauss, 1949; LeVine, 1961; Rodseth et al., 1991; Tylor, 1889). According to Chapais (2008, 2010), ‘exchanging women’ contributes to maintaining kinship bridges across residential communities. with the ability for bilateral kin and in-law recognition, and, by maintaining contact with their natal group even after dispersal, intergroup bonds (across patrilineages) can be cemented via both consanguineal kinship and affinal (in-law) kinship. Widespread exogamy (with its concomitant generation of affinal relationships) is claimed to be a strong determinant of non-warring relations among some small-scale societies, e.g. the Upper Xingu (Fry, 2006; Gregor, 1990). However, few quantitative tests of this hypothesis have been conducted, and the evidence that exogamy is sufficient to eliminate or ameliorate hostile relations is equivocal (Hayano, 1974; Kang, 1979; Kelly, 2000; see also Grueter & White, 2014). Dow et al. (2016) argue that the studies by Hayano and Kang fail to establish a positive correlation between exogamy and the absence of conflict because they do not account for the level of threat or consider alternative responses to threats. Determining the causal relationship between exogamy and intergroup conflict or tolerance is complex. Smits (2010) and Demarest and Haer (2022) show that regions with lower intermarriage rates between ethno-religious groups tend to experience more violent conflicts in Eastern Europe and Sub-Saharan Africa. However, their findings do not establish whether increasing intermarriage would actively reduce ethnic tensions or prevent armed conflict.

Contrary to the reasoning outlined in the previous paragraph, some contend that exogamy is positively correlated with war frequency (Keeley, 1996; Tefft, 1975). As Keeley (1996, p. 109) put it: “Many societies tend to fight the people they marry and to marry those they fight”. The rationale for this inference is that kinship ties fail to forge sufficiently robust bonds of mutual interest capable of averting conflicts (Tefft, 1975) or that close intergroup relationships and frequent interaction can spur dissent (Berndt, 1962; Van der Dennen, 2014).

Recently, Alami Gouraftei (2022) analyzed data from a multiethnic village at the confluence of the Amazon and Andean highlands in Bolivia to investigate whether intermarriage – marriage across ethnic boundaries – reduces group divisions and fosters minority integration and social cohesion. While intermarried individuals were well integrated into the community’s social networks and served as bridges between ethnic groups, her findings suggest that intermarriage has mixed effects on reducing negative intergroup attitudes.

The extensive kin networks created by exogamy can have knock-on utilitarian payoffs that exceed mere peace building. They allow residential mobility (visitation of kin in geographically distant areas) and access to resources in those areas in times of need. Such a safety net is particularly adaptive in hunter-gatherers that live in stochastic environments and exploit various dispersed resources (Kelly, 2013; Kramer & Greaves, 2011; Yellen & Harpending, 1972; Yengoyan, 2017; see also section below on interactions among variables).

## Cooperative Defense

“I occasionally think how quickly our differences worldwide would vanish if we were facing an alien threat from outside this world.”

— Ronald Reagan (Leuchtenburg, 2015, p. 634).

Alexander (1990) was one of the proponents of the idea that between-group competition was a driving factor in the evolution of higher-level groupings and elaborate coalitions. The introduction of a higher-level threat that requires cooperative between-group defense may thus defeat parochial sentiments and make yesterday’s enemies into today’s allies. Popular science fiction stories, such as *The War of the Worlds*, portray humanity uniting in the face of a shared existential threat. Similarly, several anthropologists (Service, 1971; Johnson & Earle, 2000; Sahlins, 1968) have argued that ‘tribes’ are, in essence, alliances of bands formed to increase competitive strength against rival groups, often in the context of resource disputes.^2^ There are several specific ethnographic examples that nicely illustrate this concept. For example, on Choiseul Island (Solomon Islands), multiple groups often formed alliances to combat similar coalitions of opposing groups, seeking retribution for perceived or actual grievances (Scheffler, 1964). And then there are the classic accounts of the Yanomamö where one group colludes with an allied group to invite a third group to a treacherous feast during which the visitors are lethally attacked (Chagnon, 1997; Helbling, 1999; Sahlins, 1968). A renowned historical example is the Iroquois Confederacy, a powerful alliance of Native American tribes that enabled its member nations to collaborate on military strategy and present a united front, particularly in response to European encroachment (Dennis, 1993). However, complete mergers are rarely the result of such alliances. In the case of the Iroquois, tribal autonomy was maintained, just as village independence remained intact among the Yanomamö (Chagnon, 2013). Extrapolating this idea of collaborative defence to larger polities, we have seen that EU member states exhibit greater cohesion and discipline when facing external threats, e.g. during confrontational relations with the Soviet bloc (Turchin & Gavrilets, 2009). Another example of a historical political transformation toward unity and peace that was driven by security interdependence is Switzerland (Fry et al., 2021). “Switzerland is a country because foreign threats forged it into one” (Parent, 2011: p. 91).

The strengthening of between-group solidarity in the face of outside threat also works at lower societal levels, e.g., clusters of individuals. For example, experiencing a shared threat, like a terrorist attack, can lead individuals who are strangers to perceive themselves as part of a cohesive social group (Drury et al., 2009). In Vanuatu, repeated dictator-game experiments before and after Cyclone Pam (2015) showed that greater exposure to people in distress post-cyclone predicted increased prosocial giving to both in-group and out-group members (Vardy & Atkinson, 2019). While external threat is probably an instrumental factor in eliciting between-group cooperation, sometimes all that is needed is an urgent superordinate goal that requires cooperative effort between groups (Brewer & Miller, 1996). The categorization of out-groups and in-groups can swiftly shift depending on shared interests (Kurzban et al., 2001). Even under conditions of shared purpose, the extension of positive regard to out-group members is not guaranteed, as they may continue to be judged through the evaluative lens of in-group norms.

The idea that external threats drive the fusion or collaboration of distinct groups is explored not only in evolutionary behavioral sciences, anthropology, and social psychology but also in fields such as cybersecurity and organizational behavior. In organizational behavior, external threats can lead to increased collaboration between separate entities. The “Fusion Doctrine” introduced by the UK government aims to improve national security by fostering cross-government collaboration in response to complex threats (HM Government, 2018).

## Interactions Among Variables

It is important to stress that these factors are correlates of peace and that the causal relationships among them are unresolved. For example, it is unclear if trade was a consequence or an initial contributor to peace. Trade and economic agreements play pivotal roles in the interactions between hunter-gatherer groups, yet they entail intricate cognitive processes and are often a result rather than the primary origin of intergroup connections (Chapais, 2013). Fry et al. (2021) also note that “it seems that economic interdependence more typically flourishes after peace systems come into being, rather than being intentionally developed at the onset as a path to peace.” (p. 8). Consequently, resource exchange de facto seems to work best when a level of tolerance and trust between groups already exists (see also Van der Dennen, 2014).

While between-group tolerance can facilitate trade relationships, trade can also strengthen or stabilize between-group tolerance, thus creating a positive autocatalytic feedback loop. Once tolerance between groups is established, it will also be easier to seek information from neighbors, intermarry, and form between-group coalitions in times of external existential threats. The xenophilic nature of between-group dynamics is often maintained by familiarity with outgroups. Social psychology has long known that previous encounters with individuals from an out-group, especially in contexts where cooperation yields positive results, can enhance an actor’s perception of a stranger belonging to the same group (Pettigrew & Tropp, 2006). A recent study combining simulations with behavioral experiments has shown that regular interactions between different groups facilitate the exchange of cooperative behaviors across group borders and foster the development of universal cooperation (Gross et al., 2023).

Trade and exogamy are not mutually exclusive, independent explanatory variables of peace. They likely interact together with alliances in potentially complex ways: exogamy may be used deliberately to establish or maintain resource exchange or alliances, and resource exchange is then used to sustain social ties between affinal relatives. For instance, Ju/’hoansi foragers benefit from marrying into families with access to different waterholes, enabling them to forage in both areas and reducing the risk of resource scarcity if one waterhole dries up (Lee, 1984). In some pastoralist systems, extensive kin networks can also be called up to ensure grazing rights in times of drought and to collectively defend herds (Bollig, 2006; Radcliffe-Brown & Forde, 1950).

Moritz et al. (2011) argues for a pastoral system that bride wealth payments associated with marriage form the foundation of trade relationships and support during a crisis. Rosenzweig & Stark (1989) analysed longitudinal data from South Indian village which strongly suggest that marriage of daughters to distant households is used as an instrument to mitigate income risks and enable consumption smoothing. And, in an ecotone in Bolivia, interethnically married couples are more resilient to the economic impacts of crop failure, probably because of enhanced variety in production approaches and access to financial assistance from extensive social support networks beyond the community (Alami Gouraftei, 2022).

Akin (1993) notes for the horticultural Kwaio in the Solomon Islands that marriages are employed strategically to form desired between-group alliances. The same argument has also been raised for the Iňupiaq Eskimos (Burch, 2005; Sheehan, 1997). And, Walker et al. (2013) more generally states for horticultural societies that exchange of marital partners and patterns of residence establish overarching social structures among groups, forging alliances that span multiple villages frequently joined in competition against other expansive alliances. Wu et al. (2023) used isotope analyses from dental enamel that indicate significant long-distance migration among females during the Longshan period (late third-millennium BC) in central China, which the authors interpret as evidence of exogamous marriage aimed at constructing political alliances. Alliances between groups/villages can also be the outcome of a combination of the exchange of women *and* trading (Chagnon, 1997). Trade in itself can also promote a web of alliances with positive repercussions for the establishment of peace structures in a region (Johnson & Earle, 2000). Finally, another activity that contributes positively to the formation of alliances between groups is mutual feasting (Chagnon, 1997; Hayden, 2014).

As touched on above, while some of the factors discussed can promote or stabilize peace between groups, they are not a recipe for perpetual peace. Peace can be a fragile construct that periodically falls apart (Keeley, 1996). In Melanesian Kwaio society, intergroup ties were sustained through intermarriage and collaborative mortuary feasts, yet these connections were intermittently disrupted by escalating cycles of blood feuds (Keesing, 1982).

## Xenophilia in our Primate Relatives

Some of our primate relatives exhibit remarkable capacity for non-agonistic, and sometimes affiliative, inter-group interactions of varying durations. These interactions can lead to the formation of amicable or cooperative relationships that extend beyond their own groups to include out-groups or individual members of other groups (Grueter & Pozzi, 2025; Grueter & White, 2014; Pisor & Surbeck, 2019). Grueter and Pozzi (2025) documented tolerant and affiliative inter-group interactions in 68 primate species. For example, certain species experience peaceful group encounters that may even involve temporary mingling, such as in western gorillas (Forcina et al., 2019), mountain gorillas (Mirville et al., 2018), and bonobos (Furuichi, 2020; Idani, 1990). In some cases, inter-group meetings include affiliative behaviors like grooming, playing, and food sharing, as observed in bonobos (Fruth & Hohmann, 2018; Samuni & Surbeck, 2023), white-handed gibbons (Reichard & Sommer, 1997), and black and gold howler monkeys (Gennuso et al., 2018). Some individuals temporarily visit neighboring groups without eliciting aggression from residents, as seen in woolly monkeys (Nishimura, 2003). Others take inter-group relationships further by engaging in cooperative behaviors, such as joint actions observed in bonobos (Sakamaki et al., 2018) and golden snub-nosed monkeys (Xiang et al., 2014). In certain species, associations between distinct social units are underpinned by coordinated activities, leading to the emergence of multi-level social systems. This form of complex social organization has been documented in several primate species such as hamadryas baboons, geladas, snub-nosed monkeys, and Rwenzori black-and-white colobus monkeys (Grueter et al., 2012, 2020; Kawai et al., 1983; Kirkpatrick & Grueter, 2010; Kummer, 1968; Miller et al., 2020; Snyder-Mackler et al., 2012; Stead & Teichroeb, 2019).

Between-group tolerance, peace, and cooperation in non-human primates may serve as functional analogues or precursors to similar phenomena in humans (Grueter, 2024; Grueter & White, 2014). For example, human exogamy, which creates supra-group kin networks, parallels dispersal patterns in primates, particularly short-distance dispersal that fosters inter-group connections. Similarly, trade and exchange in human societies represent an economic elaboration of the joint dependence on shared resources observed in primates. Furthermore, coordinated defense against out-group rivals, a hallmark of human inter-group alliances, exists in more rudimentary forms among primates.

First, we have shown that **economic and ecological interdependence** in humans – such as the trade of subsistence goods or mutually agreed-upon access to shared territories – acts as a deterrent to conflict and warfare. In primates, ecological interdependence can likewise foster inter-group tolerance, especially in species with multilevel societies. When critical resources are unevenly distributed across neighboring ranges, the incentives for exclusive access may weaken, resulting in a relaxation of inter-group tensions. This relaxed dynamic provides opportunities for greater tolerance (Jaeggi et al., 2016; Robinson & Barker, 2017) and facilitates the emergence of higher-level social structures, such as multilevel systems—so long as these structures do not impose unsustainable costs on their members (Grueter & White, 2014; see also Macdonald & Johnson, 2015).

Another advantage of extra-community relationships we discussed involves the **exchange of information** about local resource availability or improved resource acquisition strategies. While such dynamics have few direct parallels in other primates, research on bonobos has shown that intercommunity associations can enhance foraging efficiency in less familiar areas (Lucchesi et al., 2020).

We also emphasized the role of **exogamy** in mitigating underlying violent tendencies and fostering alliances by strengthening both blood and marriage-based kinship networks across groups. In primates, the development of kin networks spanning multiple units, coupled with regular opportunities for interaction between members of different groups, similarly promotes inter-group tolerance. Shared ancestry resulting from group fissions, localized dispersal, social viscosity, and extra-group matings may contribute to lower levels of aggression between neighboring groups and support the establishment of affiliative inter-group associations (Furuichi, 2020; Mirville et al., 2018; Reichard & Sommer, 1997; Rodrigues et al., 2023; Scarry & Tujague, 2012; Snyder-Mackler et al., 2014).

Lastly, as discussed above, **cooperative defense** (or aggression) against shared external threats can reduce in-group favoritism and foster amicable relationships with neighboring communities. In primates and other animal multilevel systems, such external threats – whether posed by conspecifics or predators – serve as a catalyst for developing tolerance between members of distinct units. By uniting for collective defense, groups can share the burden and mitigate the risks of external dangers (Grueter & van Schaik, 2010; Kummer, 1968; Xiang et al., 2014).

## Human Uniqueness in the Realm of Xenophilia

As shown above, some elements of xenophilia are roughly analogous to the more embryonic expression of similar phenomena in non-human primates. However, other forms of intergroup peace are exceedingly rare in non-humans. Importantly, these human-unique phenomena do not all play the same causal role. Some primarily stabilize pre-existing cooperative relationships by reducing defection and opportunism, whereas others constitute qualitatively distinct pathways by which xenophilic relations are generated, transformed or restored. In addition, some perspectives function as explanatory frameworks rather than mechanisms.

### Qualitatively Distinct Pathways

One form of friendly interaction between groups that has rarely been documented in non-humans is **peacemaking between groups**, or the ability to restore amicable relations after conflict has occurred (Garfield, 2021; see also Glowacki, 2024). Unlike mechanisms that stabilize ongoing cooperation, peacemaking operates on a different temporal axis, enabling groups to reset hostile equilibria and re-establish tolerance following breakdowns in intergroup relations. Evidence for such postconflict reconciliation at the group level is very limited in non-human primates. A study on reconciliation between social units in a golden snub-nosed monkey multilevel society failed to detect any signs of postconflict affiliation between the leader males of the core units (Zhao et al., 2016). However, in a captive hamadryas baboon colony, individuals belonging to different core units sometimes engaged in postconflict affiliation and post-conflict third-party affiliation (Romero et al., 2008; see also Butovskaya et al., 2013).

Beyond peacemaking itself, humans are uniquely capable of engaging in **diplomatic exchanges**, in which designated individuals represent group interests through negotiation, mediation, and symbolic communication (Meijer, 2024; Neumann, 2015). Diplomacy enables cooperation even in the absence of direct interpersonal ties and constitutes a pathway to intergroup tolerance that is not reducible to incentive alignment or norm enforcement.

Another distinctly human pathway is **organized intergroup play**, including ritualized competition in sports (Alexander, 1990). While such play often has a competitive – or, in the case of hooliganism, violent – component, others have emphasized its potential to reduce xenophobia and promote tolerance. Juvenile primates sometimes engage in play across group boundaries (reviewed in Grueter & Pozzi, 2025), which can foster extended social networks and reduce hostility toward strangers (Antonacci et al., 2010; Mancini & Palagi, 2009). However, unlike in humans, this play is not organized group-against-group contestation. In human societies, the integrative function of play is well illustrated by the Olympic Truce, whereby the reinstatement of the Olympic Games led warring Peloponnese regions to maintain the longest-standing peace agreement in history (Swaddling, 1999). More recently, Mousa (2020) conducted a field experiment in Northern Iraqi soccer leagues affected by ISIS violence, randomly assigning Muslim players to Christian teams and tracking behavioral changes over six months. The results showed that team-based contact improved Christian players’ inclusivity, cooperation, and recognition of Muslim teammates. Together, these findings highlight that structured intergroup play, while uniquely human in its organized form, can act as a powerful mechanism for reducing prejudice and fostering social cohesion.

**Fostering**, or the cross-group rearing of children, represents another qualitatively distinct pathway that reinforces and extends amicable relations between groups. Among the Andaman Islanders, for example, children around the age of nine or ten often left their natal households to be raised by foster parents, frequently from a neighboring group (Radcliffe-Brown). Such transfers served to consolidate and strengthen social bonds between the birth family and the foster family (Donner, 1999; Goody, 1982). In humans, therefore, fostering can operate as a deliberate social strategy to sustain intergroup alliances. By contrast, cross-group adoption is exceedingly rare among nonhuman primates, with cases reported in colobus monkeys (Dunham & Opere, 2016), titi monkeys (Cäsar & Young, 2008), and bonobos (Tokuyama et al., 2021). In these instances, adults provided care to extra-group infants, including nursing, carrying, grooming, and food sharing. However, unlike human fostering, there is little evidence that such adoptions function to promote or sustain friendly relations between groups.

Another mechanism for fostering collective identity is **joint music-making and synchronized movement**, which can enhance social closeness and encourage cooperative behaviors, even in large groups of strangers and across intergroup boundaries (Good et al., 2017; Harvey, 2017; Weinstein et al., 2016). These effects operate through affective and physiological mechanisms rather than through the stabilization of existing cooperative dilemmas, offering yet another distinct proximate route to xenophilia. History and contemporary practice provide striking illustrations of this effect, from spontaneous carol singing between enemy soldiers during the 1914 Christmas Truce to cross-cultural initiatives like Daniel Barenboim’s West-Eastern Divan Orchestra, which brings together young Israeli, Palestinian, and Arab musicians to promote dialogue and trust (see e.g. Harvey, 2017).

### Stabilizers of Cooperative Dilemmas

Other human-unique mechanisms primarily function to stabilize intergroup cooperation once interaction has been established. A recent target article in *Behavioral and Brain Sciences* (Glowacki, 2024) has stressed that sustained peaceful relationships in human societies are difficult to achieve because they require solving a **cooperative dilemma**. Some self-interested individuals may benefit more from engaging in aggressive or exploitative behaviour toward members of other groups than from maintaining harmony, even when such behaviour is collectively damaging (Cohen & Insko, 2008). As Glowacki (2024) notes, “the difficulty of limiting the payoffs of aggression by individuals is one of the most formidable barriers to the emergence of peace” (p. 5).

This vulnerability is particularly acute in the context of intergroup and long-distance relationships, where opportunities for monitoring and immediate reciprocation are limited. As emphasized by (Tooby & Cosmides, 1996) “Banker’s Paradox,” individuals or groups most in need of assistance – such as during resource shortfalls, conflict or ecological shocks – are often least able to reciprocate in the future, making such relationships especially prone to exploitation. In the absence of additional safeguards, xenophilic ties would therefore be expected to be unstable and easily undermined by freeriding.

Humans appear to have addressed this problem through the development of **non-warring cultural norms and institutions** that prescribe prosocial conduct toward other groups and govern the terms of exchange (Fearon & Laitin, 1996; Fry et al., 2021; Glowacki, 2024; Robinson & Barker, 2017). By limiting the payoffs of aggression and sanctioning norm violators, such institutions help solve the collective action problems inherent in sustained peace systems. These cultural norms, do, however, vary across societies. Individualistic cultures (e.g., Western cultures) often support cooperation based on universal principles, while collectivist cultures (e.g., East Asian cultures) prioritize in-group loyalty (Triandis, 2018; Yamagishi et al., 1998).

Relatedly, **reputational incentives and impression management** can motivate individuals and groups to behave cooperatively toward outsiders in order to enhance collective status or credibility (Leeuwen & Mashuri, 2012). Here, helping is used strategically to present one’s own group in a positive light, and the resulting boost in status or reputation can be adaptive. These mechanisms raise the costs of defection and reinforce peaceful equilibria rather than generating new ones.

At a broader social scale, institutionalized social structures such as age-set systems, lineal kin groups and segmentary lineages can also facilitate durable cross-group and long-distance ties, coordinating behavior, enforcing norms and creating cross-cutting obligations. In several small-scale societies, these structures create cross-cutting obligations, shared identities and coordination mechanisms that extend beyond local communities and help regulate cooperation and conflict at broader spatial scales (Glowacki, 2020). Such systems can therefore be understood as additional social technologies that stabilize and scale peaceful intergroup relations by supporting commitment, trust and third-party coordination across spatially dispersed groups.

Societies or communities that live in harmony and/or engage in exchange often use **fictive kinship**, i.e., the use of kinship terms and metaphors to describe non-relatives, to project familial feelings of closeness to unrelated extra-group members, thereby expanding their network of relationships (Hruschka, 2010; Kandza et al., 2023). While fictive kinship can also contribute to identity transformation, its primary role in many contexts is to stabilize existing cooperative networks. For example, in the Congo Basin, fictive kinship coordinates the exchange of goods acquired by foragers (e.g., honey, medicine) with those available in farming villages (e.g., manioc, iron) and connects individual foragers to individual farmers, and by extension unites their families (Joiris, 2003). Similarly, among the Iroquoians, the strategic use of kinship terminology and imagery fostered a shared pan-Iroquois identity, strengthening alliances across multiple groups (Dennis, 1993; Fry, 2012). These systems demonstrate how fictive kinship, embedded in broader cultural practices, can create durable networks that bridge social divisions. By contrast, it is unclear whether a single linguistic cue can achieve a similar effect in transient, modern interactions. One such cue is the Australian term *mate*, associated with mateship, a tradition emphasizing loyalty and mutual support (Dyrenfurth, 2015). To test whether such affiliative language can bridge divides between groups, Grueter et al. (2021) conducted an experiment in which listeners rated the trustworthiness of speakers whose voices varied by accent (Australian English, British English, or foreign-accented English) and by the presence or absence of the term ‘mate.’ As expected, listeners trusted speakers with their own dialect more, but the inclusion of mate did not increase trustworthiness, suggesting that isolated affiliative terms may be insufficient to expand social networks across sociolinguistic boundaries.

**Religious beliefs and practices** can play a dual role in intergroup relations. Insofar as they promote inclusive moral norms and regulate behavior toward out-groups, they function as stabilizers of cooperation by limiting defection and reinforcing prosocial expectations. At the same time, when religious identities transcend group boundaries and reconfigure social categories, they can operate as distinct pathways to xenophilia by fostering more encompassing forms of social identification (Ginges et al., 2016; Northover et al., 2024). High-cost, infrequently performed, and highly collective religious practices—such as the Hajj or the kavadi ritual—have been linked to the development of more inclusive social identities and greater openness toward out-group members (Northover et al., 2024). Moreover, many religious traditions explicitly promote cooperative engagement across group boundaries, and activation of religious beliefs can motivate adherents to extend prosocial behaviors beyond their immediate in-group (Ginges et al., 2016; Pasek et al., 2023; Van der Dennen, 2014). 

These stabilizing mechanisms raise a broader comparative question: how are analogous cooperative dilemmas managed in non-human primates, which lack formal norms and institutions? One possibility is through processes such as sharing or decentralized collective decision-making (Hunt et al., 2024). Interestingly, the existence of a collective action problem has also been invoked to explain the generally low intensity of between-group aggression observed in some primate species. According to Willems & van Schaik (2015), effective collective action against out-groups requires the suppression of freeriding, which may constrain the escalation of intergroup violence.

### Explanatory Frameworks

Finally, some approaches invoked to explain human intergroup cooperation do not constitute mechanisms but rather evolutionary explanatory frameworks. One of these that has been applied to explain cooperation between groups in humans, but is unlikely to be of relevance to non-humans, is **cultural group selection**, i.e. the process by which populations with group-beneficial cultural traits outcompete other groups (Smith, 2020). As stated by Zefferman and Mathew (2015), group-structured cultural selection predicts that norms for warfare are more likely to be found along social boundaries characterized by cultural differentiation whereas peace is more likely to occur along social boundaries with little cultural differentiation. In support of this model, Handley and Mathew (2020) find that among pastoralists of NW Kenya cooperation across social boundaries is predicted by how culturally similar they are. Bonobos may represent one non-human example where cultural distinctiveness between groups in some domains (prey preferences) does NOT erode between-group tolerance (Samuni et al., 2020, 2024).

## Conclusions

In summary, peaceful interactions between human groups provide numerous advantages. Several key factors have been identified in fostering cooperative intergroup relationships and broadening the scope of tolerance, including economic interdependence through trade and reciprocity, knowledge sharing, exogamous marriages, and the necessity of collective defense against external threats. While similar socioecological dynamics exist in some non-human primates, they lack the cultural reinforcement observed in human societies. In humans, symbolic systems such as fictive kinship, shared religious practices and institutionalized norms further support the persistence of intergroup peace by regulating conflict and curbing self-serving aggression.

Taken together, the mechanisms reviewed in this paper play distinct but complementary roles in sustaining peaceful intergroup relations. Some processes – such as trade, exogamy, and shared defense – generate incentives for cooperation, while others primarily function to stabilize these relationships by limiting defection, aggression, and freeriding through norms, institutions, reputational incentives and symbolic practices. Still other human-unique processes, including peacemaking, diplomacy, organized intergroup play, fostering and synchronized ritual, constitute qualitatively distinct pathways to xenophilia by transforming social identities, emotional alignment, or post-conflict dynamics. Recognizing these differences helps clarify how diverse mechanisms jointly contribute to the emergence, maintenance and recovery of intergroup peace. Overall, these findings suggest that while the cognitive and social foundations of xenophilia are widespread features of our species, their expression, stability and scale depend on stabilizing mechanisms that vary systematically with ecology, subsistence, market integration and institutional context.

Several of the patterns reviewed here generate clear, testable predictions for future work, illustrating how the synthesis can be extended in a hypothesis-driven direction. For example, intergroup tolerance and exchange ties should be strongest where resource shortfalls are spatially asynchronous rather than synchronized, conditional on population density and mobility constraints. Peaceful relations should also be more stable where intergroup ties are multiplex – combining trade, kinship, ritual participation, or alliance – than where ties are single-stranded, because multiplexity raises the opportunity costs of conflict and increases opportunities for mediation. Finally, the persistence of peace systems should depend not only on ecological incentives for cooperation but also on the capacity for norm enforcement, whether through institutions, reputational mechanisms or culturally shared peace norms. These predictions highlight how ecological payoffs, relational structure and enforcement capacity jointly shape the emergence, stability, and breakdown of xenophilic relationships.

Understanding inter-group tolerance is crucial for unraveling the emergence of highly interconnected social networks that facilitated the exchange of innovative ideas and spurred technological revolutions through cultural recombination and accumulation (e.g. Derex & Boyd, 2015; MacDonald et al., 2021; Powell et al., 2009; Spikins et al., 2021), These dynamics have significantly contributed to the evolutionary success of our species. Moreover, exploring the evolutionary roots of tolerance and cooperation across group boundaries remains a pressing priority for addressing global challenges such as climate change, poverty, famine, conflict, and disease. In the face of climate change, social investments aimed at buffering resource shortfalls through extra-group tolerance and exchange may become increasingly important (Pisor & Jones, 2021). More generally, the framework developed here highlights how recurrent human capacities for xenophilia, when coupled with stabilizing social mechanisms, can generate durable intergroup peace across a wide range of ecological and social conditions.

## Data Availability

No datasets were generated or analysed during the current study.

## Footnotes

1 A related perspective is offered by the Founder Sociality Hypothesis (Brooks & Yamamoto, 2021), which posits that when populations colonize new or newly available environments, ambiguous territorial boundaries, encounters with unfamiliar individuals, and initial resource abundance can favor reduced territoriality, increased social tolerance, and novel bonding patterns, potentially producing long-lasting shifts in social organization.

2 A similar dynamic has been invoked to explain larger-scale political transformations. Carneiro (1970) argued that competition over productive land drove chiefdoms into multi-tribal alliances.
